# Prevalence, severity, and correlates of erectile dysfunction among male adult patients of a primary care clinic in North-West Nigeria

**DOI:** 10.4314/ahs.v23i2.77

**Published:** 2023-06

**Authors:** Abdullahi Z Muhammad, Bukar A Grema, Abdulrahman Shuaibu, Godpower C Michael

**Affiliations:** 1 Department of Family Medicine, Federal Medical Centre, Azare, Bauchi State, Nigeria; 2 Department of Family Medicine, Aminu Kano Teaching Hospital, Kano, Nigeria

**Keywords:** Correlates, erectile dysfunction, obesity, primary care, severity, Northwest Nigeria

## Abstract

**Background:**

Erectile dysfunction (ED) is a common sexual problem that profoundly affects intimate relationships. It is poorly studied in North-western Nigeria.

**Objectives:**

To assess the prevalence, severity and predictors of ED among adult males attending a primary care clinic in Northwest Nigeria.

**Methods:**

A cross-section of 392 males (aged ≥25 years) were randomly selected from attendees of a family medicine clinic in Kano, Nigeria. Information regarding their biodata, lifestyle factors, and clinical characteristics was obtained using a structured questionnaire. The International Index of Erectile Function Questionnaire (version 5) and Patient Health Questionnaire-2 assessed ED and depression, respectively.

**Results:**

The participants' mean age was 45±14.1 years (range: 26-86 years). Most participants were married (88.8%), had one sexual partner (71.7%), had tertiary education (44.4%) and were traders (49.7%). The prevalence of ED was 52.3% (205/392) [comprising mild (36.0%), mild-to-moderate (14.3%), moderate (1.5%) and severe (0.5%)]. Age, body mass index (BMI), marital status, number of sexual partners, monthly income, type of exercise, hypertension history, blood pressure reading, antihypertensive and peptic ulcer drug use were associated with ED (P<0.05). However, age (≥50years) (OR= 1.91, 95%CI [1.28-2.85], P=0.001) and overweight/obesity (OR =1.81, 95%CI [1.18-2.77], P=0.007) were the predictors of ED in this population.

**Conclusion:**

ED prevalence was high, although mainly of the mild form. Age (≥50years) and overweight/obesity predicted ED. Hence, the need for early screening, objective grading of ED, identification of modifiable risk factors (e.g., overweight/obesity) and commencing proper treatment and prevention in this setting.

## Introduction

Sexual health and function are important determinants of quality of life.^1^ Erectile dysfunction (ED), the persistent inability to attain and/or maintain an erection adequate for satisfactory sexual intercourse for over three months, is a common male sexual dysfunction of public health importance given its comorbidities, preventive risk factors, the increasing proportion of the elderly and newer management approaches.^2^,^3^,^4^ An estimated 152 million men worldwide have some degree of erectile dysfunction (ED); it is projected that the number will rise to 322 million by 2025.^5^ Although the prevalence increases with increasing age, ED prevalence also varies with the study setting and the assessment tool used (the single National Institute of Health [NIH] consensus questionnaire tends to yield higher prevalences than the International Index of Erectile Function (IIEF-5) Questionnaire).^5^ Prevalences of 49.4%, 34%, 63.6%, 80.8% has been reported in primary care settings of Canada, Australia, Egypt and Pakistan, respectively.^6^,^7^,^8^ Similarly, in Nigeria, few studies on ED have been reported, predominantly in the southern geopolitical zones of Nigeria. ED prevalences of 57.4% (in a multi-country national survey in primary care), 55.1% (in a primary care clinic in Ibadan, southwestern Nigeria), 41.5% (in a primary care setting in Calabar, South-south geopolitical zone), 43.8% (in a community-based study in Osogbo, south-west Nigeria), 46.9% (community-based study in Ilorin, North-central), and 58.9% (community-based study in Ogbomoso, South-west) have been reported.^3^,^8^-^12^ Similar studies in the North-west geopolitical zone of Nigeria are scarce.

Furthermore, although racial biases have been reported, unhealthy lifestyles, poverty, and high prevalence of chronic medical conditions increase the prevalence of ED among blacks compared to Caucasians.^13^,^14^ However, ED is under-estimated in developing countries (Nigeria inclusive); this has been attributed to poor help-seeking behaviour among patients because they consider ED non-life-threatening, a social stigma, or are shy, ignorant, and reluctant to confide their private matters with their doctor. 3,9,10,15,16 This is worrisome at a time when ED is not only regarded as a correlate of cardiovascular diseases, diabetes and metabolic syndrome but could be an early warning symptom and a marker for disease progression.^17^ Fortunately, many ED risk factors are preventable, necessitating the screening of ED and its correlates in the primary care setting where a preponderance of citizens interface with the health care system.

This study, therefore, assessed the prevalence, severity and predictors of ED in male adult patients attending a primary care clinic in North-western Nigeria. It is hoped that the findings will provide valuable data on ED in Northwest Nigeria that could support screening in primary care patients for prompt intervention.

## Materials and Methods

### Study design, setting and population

This descriptive cross-sectional study was carried out between September to November 2015 at the General Outpatient Clinic (GOPC) of a Nigerian tertiary hospital. The hospital is located in the capital of the State. The State is located in the North-West geopolitical zone of Nigeria. The inhabitants of the State are mainly traders and civil servants. They are predominantly of the Hausa and Fulani tribes and the Islamic faith. The Igbo, Yoruba, Tiv, Nupe, Ebira, Kanuri ethnic groups are also residents. The hospital is a 500-bed hospital serving the host state and neighbouring states as a referral centre. In addition, the hospital has specialist clinics that consult patients on referral. A large proportion of patients are therefore managed at GOPC. The GOPC is a busy primary care centre run by family physicians, family medicine residents and general duty doctors. The clinic records show that an average of 250 undifferentiated adult male 43% (108/250) and female 57% (142/250) patients are seen daily on workdays. The study population consisted of male adult patients aged ≥25 years who came to the GOPC of the hospital for consultation.

### Eligibility criteria

Consenting male adult patients (aged ≥25 years) who came to the GOPC for consultation were included in this study. However, those who required urgent medical attention, had a profound mental illness, a previous diagnosis of ED, penile prosthetic devices, or had not attempted sexual activity six months before the study were excluded.

### Sample size estimation

The sample size was calculated using the Fisher's formula (n = Zα2 p q/ d2),18 where n = minimum sample size, Zα = standard deviation corresponding to the 95% confidence interval (1.96), p = prevalence rate (55.1%, prevalence rate of ED from a previous study in Ibadan, Nigeria),11 q = 1 -- p, and d = level of precision, usually set at 5%. Thus, a minimum sample size of 380 was obtained, but an additional 10% (38) were added to accommodate non-responses, attrition and missing data to give a sample of 418.

### Sampling method

A list of male adults was made at the triage unit. Systematic random sampling was used to recruit 418 participants for this study. An average of 540 (108x5) male adult patients is seen weekly in the GOPC during workdays. With a study duration of 12 weeks, the sampling frame was 12 x 540 = 6480. The sampling interval of 15 was calculated by dividing the sampling frame by the sample size (6480/418 ≈ 15). Hence, every 15^th^ patient on the list, who met inclusion criteria, was recruited after the first participant was selected by balloting from the first 15 listed patients; this continued until the sample size was reached.

### Data collection

An investigator-administered questionnaire in English and Hausa languages was used to collect data. The questionnaire, initially in English, was translated into Hausa (the local language spoken by the majority of patients) by a Hausa linguistic professional experienced in health surveys. Precise idiomatic equivalents were employed as much as possible. The back translation, performed independently by another linguist, was compared with the original version and confirmed satisfactory before pretesting. The questionnaire was pretested at the general outpatient clinic of Murtala Muhammed Specialist Hospital, Kano, using thirty participants. The questionnaire consisted of the following sections: sociodemographic characteristics; lifestyle factors (smoking, alcohol, physical activity); clinical correlates (hypertension, obesity, diabetes, dyslipidemia, medications, previous pelvic surgery); Patient Health Questionnaire-2 (PHQ-2)^19^ and International Index of Erectile Function (IIEF-5).^11^,^12^,^20^

### Definition and measurement of variables

The primary outcome variable was erectile dysfunction. Using the IIEF-5 questionnaire, ED was classified with scores of 1 to 25: scores of 1-7 (severe ED), 8-11 (moderate ED), 12-16 (mild to moderate ED), 17-21 (mild ED) and 22-25 (no ED). The PHQ-2 was used to screen for depression; it inquires about the frequency of depressed mood and anhedonia over the past two weeks; if the participant's response to both questions is “no”, the screen is negative. If the patient responded “yes” to either question, there is a high likelihood of depression.^19^

Diabetes was defined as a fasting plasma glucose of ≥ 7mmol/L. Dyslipidemia was defined as serum total cholesterol of ≥5.17mmol/L, triglyceride of ≥1.7mmol/L, LDL cholesterol of ≥2.58mmol/L and HDL cholesterol of <1.03mmol/L.^21^

### Study protocol

Potentially eligible patients were given information by a trained research assistant at the triage unit of the GOPC on the research objectives, procedure, and their rights to refuse participation prior to recruitment. Written informed consent was obtained from potential participants while in the waiting area. The participants were then assigned to a designated consulting room. The principal investigator completed the questionnaire in the consulting room; the primary reason for the clinic visit was also addressed. Similarly, the participant's height (in meters), body weight (in kilograms) and blood pressure (in mmHg) were measured.

Participants were asked to return the next day after an overnight fast (8-12 hours) for a fasting blood glucose and lipid profile;3mls of venous blood was collected in a Fluoride Oxalate bottle for fasting plasma glucose, and 4mls of blood was collected in a Lithium Heparin bottle for lipid profile. In addition, a urine sample was collected in a sterile specimen bottle and tested for glucosuria, proteinuria and nitrites.

### Ethical considerations

Ethical approval (NHREC/21/08/2008/AKTH/EC/1188) was obtained from the medical research ethics committee of the hospital. Information on the study objectives and procedure were given before written informed consent was obtained from each participant. The participants were reassured of the confidentiality of all information provided, and all questionnaires were de-identified. All participants were educated and counselled by the researcher on erectile dysfunction and its correlates. They also received information on regular exercise, healthy eating, smoking and, where necessary, smoking cessation. In addition, participants with abnormal urinalysis, elevated blood pressure, fasting blood glucose, and BMI were counselled and further evaluated. Those identified with ED or any of its correlates were managed accordingly with adequate patient education on lifestyle interventions (smoking cessation, alcohol cessation, weight reduction and exercise), counselling, drug therapy, proper management of associated clinical conditions and referral to the urologist.

### Data analysis

Data were entered and analysed using the Statistical Package for the Social Sciences version 17 software. Absolute numbers and simple percentages were used to describe categorical variables. Similarly, quantitative variables were described using measures of central tendency (mean) and measures of dispersion (range, standard deviation) as appropriate. Frequencies and proportions were used to describe the characteristics of the study population. The Chi-square or Fisher's test (as applicable) was used to assess the significance of association between categorical variables. A p-value of < 0.05 was considered statistically significant. The predictors of ED were determined by logistic regression of variables that were significantly associated with erectile dysfunction on bivariate analysis.

## Results

### Sociodemographic characteristics of participants

A total of 418 participants were recruited during the study period. However, 17 did not return for the follow-up investigation, six had grossly incomplete questionnaires, while three did not return with investigation results; thus, the data of 392 participants (representing a response rate of 93.8%) were analysed. Table 1 shows the sociodemographic characteristics of the participants. Their mean age was 45 ± 14.1 years (range: 26-86 years). Most participants were married (348, 88.8%), resided in urban areas (320, 81.6%), had one sexual partner (281, 71.7%), were Muslims (349, 89.0%), had tertiary education (174, 44.4%), were traders (195, 49.7%) and earned ≥18000 naira monthly (320, 81.6%).

**Table 1:** Sociodemographic characteristics of participants (n=392)

Characteristics	n (%)
**Age (years) [mean = 45 ± 14.1 years]**	
18-29	24 (6.1)
30-39	116 (29.5)
40-49	81 (20.7)
50-59	79 (20.2)
≥60	92 (23.5)
**Residence**	
Urban	320 (81.6)
Rural	72 (18.4)
**Marital status**	
Single	25 (6.4)
Married	348 (88.8)
Separated / divorced	15 (3.8)
Widowed	4 (1.0)
**No. of sexual partners**	
1	281 (71.7)
2	93 (23.7)
>2	18 (4.6)
**Religion**	
Islam	349 (89.0)
Christianity	43 (11.0)
**Tribe**	
Hausa	309 (78.8)
Fulani	20 (5.1)
Yoruba	10 (2.6)
Igbo	20 (5.1)
Others	33 (8.4)
**Educational level**	
No formal education	114 (29.1)
Primary	58 (14.8)
Secondary	46 (11.7)
Tertiary	174 (44.4)
**Occupation**	
CS	112 (28.6)
Traders	195 (49.7)
Self employed	35 (8.9)
Retired CS	32 (8.2)
Unemployed	18 (4.6)
**Monthly income (₦)**	
<18,000	72 (18.4)
≥18,000	320 (81.6)

### Prevalence and pattern of erectile dysfunction among participants

Most participants, 205 (52.3%), had various forms of ED, while 187 (47.7%) had no ED. Among those with ED, 141 (36.0%) had mild ED, 56 (14.3%) had mild to moderate ED, 6 (1.5%) had moderate ED, and 2 (0.5%) had severe ED.

### Participants' medical conditions

Table 2 shows the background medical condition of the participants. Most participants had hypertension (57.9%); 44.6% were on antihypertensive medication; 11.7% had diabetes mellitus, but 3.8% were on oral hypoglycaemic agents; 10.7% had peptic ulcer disease, and 9.9% were on peptic ulcer disease medications.

**Table 2:** Participants' medical condition and erectile dysfunction

Characteristics	n (%)	Erectile dysfunction, n (%)		Total	Test statistics
		Yes	No	n (%)	
**History of hypertension**					
Yes	227 (57.9)	155 (68.3)	72 (31.7)	227 (100)	P=0.000*
No	165 (42.1)	50 (30.3)	115 (60.7)	165 (100)	χ^2^=55.24
**Antihypertensive use**					
Yes	175 (44.6)	124 (70.9)	51 (29.1)	175 (100)	P=0.000*
No	217 (55.4)	81 (37.3)	136 (62.7)	217 (100)	χ^2^ =43.66
**Diabetes**					
Yes	46 (11.7)	30 (65.2)	16 (34.8)	46 (100)	P=0.0.062
No	346 (88.3)	175 (50.6)	171 (49.4)	346 (100)	χ^2^ =3.49
*OHA use*					
Yes	15 (3.8)	8 (53.3)	7 (46.7)	15 (100)	P=0.935
No	377 (96.2)	197 (52.3)	180 (47.7)	377 (100)	χ^2^=0.007
*CKD*					
Yes	1 (0.3)	1 (100)	0	1 (100)	P=0.523^f^
No	391 (99.7)	204 (52.2)	187 (47.8)	391 (100)	
**Dyslipidaemia**					
Yes	6 (1.5)	2 (33.3)	4 (66.7)	6 (100)	P=0.300
No	386 (98.5)	203 (52.6)	183 (47.4)	386 (100)	χ^2^=0.88
*PUD*					
Yes	42 (10.7)	16 (38.1)	26 (61.9)	42 (100)	P=0.610
No	350 (89.3)	189 (54.0)	161 (46.0)	350 (100)	χ^2^ =3.80
**PUD medication use**					
Yes	39 (9.9)	12 (30.8)	27 (69.2)	39 (100)	P=0.005*
No	353 (90.1)	193 (54.7)	160 (45.3)	353 (100)	χ^2^=8.05
Medication use** duration (years)					
<1	17 (7.4)	9 (52.6)	8 (47.4)	17 (100)	P=0.061
1-5	89 (38.7)	46 (51.6)	43 (48.4)	89 (100)	χ^2^=16.07
6-10	82 (35.6)	57 (69.9)	25 (30.1)	82 (100)	
>10	42 (18.3)	35 (84.1)	7 (15.9)	42 (100)	
**Lower abdominal/groin surgery**					
Yes	8 (2.0)	6 (75.0)	2 (25.0)	8 (100)	P=0.288^f^
No	384 (98.0)	199 (51.8)	185 (48.2)	384 (100)	

OHA: Oral hypoglycaemic agent

* Significant

f Fisher's exact test

** only single medication use was compared

### Participants' lifestyle practices

Only a few participants were current smokers (2.0%), using cannabis (0.3%) and taking alcohol (3.8%) (Table 3). However, 177 (45.2%) engaged in exercise, mostly brisk walks (98, 55.4%), but more exercised <3 times per week (74, 41.8%) for <30 minutes (75, 42.4%).

**Table 3:** Participants' lifestyle practices and erectile dysfunction

Characteristics	N (%)	Erectile dysfunction, n (%)		Total	P value
		Yes	No	n (%)	
**Cigarette smoking**					
Never smoked cigarette	341 (87.0)	173 (50.7)	168 (49.3)	341 (100)	0.199^f^
Current smoker	8 (2.0)	4 (50)	4 (50)	8 (100)	
Quit	43 (11.0)	28 (65.1)	15 (34.9)	43 (100)	
**Duration since quitting smoking (years) n=43**					
= 10	17 (39.5)	9 (52.9)	8 (47.1)	17 (100)	0.957
>10	26 (60.5)	19(52.3)	7(47.7)	26 (100)	
**Cannabis use**					
Yes	1 (0.3)	0	1 (100)	1 (00)	0.477^f^
No	391 (99.7)	205 (52.4)	186 (47.6)	391(100)	
**Alcohol intake**					
Yes	15 (3.8)	5 (33.3)	10 (66.7)	15 (100)	0.187
No	377 (96.2)	200 (53.1)	177 (46.9)	377 (100)	
**Exercise**					
Yes	177 (45.2)	98 (55.4)	79 (44.6)	177 (100)	0.269
No	215 (54.8)	107 (49.8)	108 (50.2)	215 (100)	
**Type of exercise (n = 177)**					
Brisk walking	98 (55.4)	67 (68.7)	31 (31.3)	98 (100)	< 0.001*^f^
Jogging	57 (32.2)	26 (45.6)	31 (54.4)	57 (100)	
Racket ball	1 (0.5)	0	1 (100)	1 (100)	
Football	21 (11.9)	5 (19)	16 (81)	21 (100)	
**Exercise frequency (n= 177)**					
Daily	26 (14.7)	19 (73.1)	7 (26.9)	26 (100)	0.212
> 3 times / week	14 (7.9)	7 (50)	7 (50)	14 (100)	
3 times/ week	63 (35.6)	35 (55.6)	28 (44.4)	63 (100)	
< 3 times / week	74 (41.8)	36 (49.3)	38 (50.7)	74 (100)	
**Exercise duration (n= 177)**					
< 30 minutes	75 (42.4)	45 (60)	30 (40)	75 (100)	0.08
30 minutes	41 (23.2)	26 (63.4)	15 (36.6)	41(100)	
> 30 minutes	61 (34.4)	27 (43.5)	34 (56.5)	61 (100)	

f Fisher's exact test

* Significant

### Participants' clinical characteristics

More participants had normal BMI (201, 51,2%), prehypertension (166, 42.4%) and normal fasting blood glucose (345, 88.0%) (Table 4). However, a few had a positive depression screening (16, 4.1%) and glucosuria (19, 4.9%).

**Table 4:** Participants' clinical characteristics and erectile dysfunction

Characteristics	N (%)	Erectile dysfunction, n (%)		Total	P-value
		Yes	No	n (%)	
**Body mass index (BMI)**					
Underweight	7 (1.8)	2 (28.6)	5 (71.4)	7 (100)	< 0.001*^f^
Normal	201 (51.2)	86(42.8)	115(57.2)	201(100)	
Overweight	141 (36.0)	84 (59.6)	57 (40.4)	141(100)	
Obese	43 (11.0)	33 (76.7)	10 (23.3)	43 (100)	
**Blood pressure**					
Normal	77 (19.6)	26 (33.8)	51 (66.2)	77 (100)	< 0.001*
Pre-hypertension	166 (42.4)	67 (40.4)	99 (59.6)	166 (100)	
Stage 1	92 (23.5)	67 (72.8)	25 (27.2)	92 (100)	
Stage 2	57 (14.5)	45 (78.9)	12 (21.1)	57 (100)	
**Fasting blood glucose**					
Normal	345 (88.0)	175 (50.7)	170 (49.3)	345 (100)	0.061
Impaired	19 (4.9)	8 (42.1)	11 (57.9)	19 (100)	
Elevated	28 (7.1)	22 (78.6)	6 (21.4)	28 (100)	
**Dyslipidaemia**					
Yes	310 (79.1)	161 (52.1)	149 (47.9)	310(100)	0.873
No	82 (20.9)	43 (53.1)	39 (46.9)	82(100)	
**Depression screening**					
Positive	16 (4.1)	9(56.2)	7 (43.8)	16(100)	0.803
Negative	376 (95.9)	196(52.1)	180 (47.9)	376(100)	
**Urinalysis**					
Negative	359 (91.6)	185(51.5)	174 (48.5)	359 (100)	0.331^f^
Glucosuria	19 (4.9)	12 (63.2)	7 (36.8)	19 (100)	
Proteinuria	8 (2.0)	6 (75)	2 (25)	8 (100)	
Nitrite	6 (1.5)	2 (33.3)	4 (66.7)	6 (100)	

* Significant

f Fished's exact test

### Association of sociodemographics and pattern of erectile dysfunction among participants

The proportion of participants with various forms of ED increased with increasing age (Table 5). This association was statistically significant (Fisher's exact, P<0.001). Similarly, more married participants had various forms of ED than the other marital status groups; also, more participants had mild ED (39.1%) than the other ED forms (15.5%-mild to moderate, 1.7%-moderate, 0.3%-severe ED); these associations were statistically significant (Fisher's exact, P=0.001). Furthermore, the proportion of participants with various forms of ED increased with an increasing number of sexual partners; this association was statistically significant (Fisher's exact, P<0.001). Except for mild ED, the prevalence of the various forms of ED was higher in participants who earned <₦18000 monthly than those who earned ≥ ₦18000; this association was statistically significant (Fisher's exact, P<0.003). However, their area of residence, religion, educational level and occupation were not significantly associated with the ED pattern (P>0.05).

**Table 5:** Association between participants' sociodemographic and pattern of erectile dysfunction

Characteristics	Erectile dysfunction n (%)					Test statistics
	No	Mild	Mild to moderate	Moderate	Severe	
**Age (years)**						
18-29	20 (83.3)	3 (12.5)	1 (4.2)	0	0	P<0.001*^f^
30-39	78 (67.2)	29 (25)	9 (7.8)	0	0	
40-49	43 (53.1)	28 (34.6)	10 (12.3)	0	0	
50-59	25 (31.6)	41 (51.9)	12 (15.2)	1 (1.3)	0	
≥60	21 (22.8)	40 (43.5)	24 (26.1)	5 (5.4)	2 (2.2)	
**Residence**						
Urban	154 (48.1)	115 (35.9)	44 (13.8)	5 (1.6)	2 (0.6)	P=0.944^f^
Rural	33 (45.8)	26 (36.1)	12 (16.7)	1 (1.4)	0	
**Marital status**						
Single	22 (88.0)	2 (8.0)	1 (4.0)	0	0	P=0.001*^f^
Married	151 (43.4)	136 (39.1)	54 (15.5)	6 (1.7)	1 (0.3)	
Separated /divorced	12(80.0)	3 (20.0)	0	0	0	
Widowed	2 (50.0)	0	1 (25.0)	0	1 (25.0)	
**No. of sexual partners**						
1	156 (55.5)	93 (33.1)	29 (10.3)	3 (1.1)	0	P<0.001*^f^
2	27 (29)	39 (43)	22 (23.7)	3 (3.2)	1 (1.1)	
>2	4 (22.2)	8 (44.4)	5 (27.8)	0	1 (5.6)	
**Religion**						
Islam	166 (47.6)	122 (35)	54 (15.5)	5 (1.4)	2 (0.6)	P=0.320^f^
Christianity	21 (48.8)	19 (44.2)	2 (4.7)	1 (2.3)	0	
**Tribe**						
Hausa	149 (48.2)	110 (35.6)	44 (14.2)	4 (1.3)	2 (0.6)	
Fulani	7 (35.0)	4 (20.0)	8 (40.0)	1 (5.0)	0	
Yoruba	5 (50.0)	3 (30.0)	2 (20.0)	0	0	
Igbo	8 (40.0)	11 (55.0)	0	1 (5.0)	0	
Others	18 (54.5)	13 (39.4)	2 (6.1)	0	0	
**Educational level**						
No formal education	45 (39.5)	44 (38.6)	23 (20.2)	2 (1.8)	0	P=0.311^f^
Primary	31 (53.4)	23 (39.7)	4 (6.9)	0	0	
Secondary	23 (50.0)	16 (34.8)	6 (13.0)	1 (2.2)	0	
Tertiary	88 (50.6)	58 (33.3)	23 (13.2)	3 (1.7)	2 (1.1)	
**Occupation**						
CS	61 (54.5)	35 (31.2)	16 (14.3)	0	0	P=0.101^f^
Traders	91 (46.7)	78 (40.0)	25 (12.8)	1 (0.5)	0	
Self employed	14 (40.0)	14 (40.0)	7 (20.0)	0	0	
Retired CS	10 (31.2)	10 (31.2)	7 (21.9)	3 (9.4)	2 (6.2)	
Unemployed	11 (61.1)	4 (22.2)	1 (5.6)	2 (11.1)	0	
**Monthly income (₦)**						
<18,000	30 (41.7)	22 (30.6)	14 (19.4)	5 (6.9)	1 (1.4)	P=0.003*^f^
≥18,000	157 (49.1)	119 (37.2)	42 (13.1)	1 (0.3)	1 (0.3)	

* Significant

f Fisher's exact test

CS: Civil servant

### Predictors of erectile dysfunction among the participants

On bivariate analysis, a history of hypertension, antihypertensive and peptic ulcer disease medication use (Table 2), type of exercise they engaged in (Table 3), their BMI, blood pressure reading (Table 4), age, marital status, number of sexual partners, and monthly income (Table 5) had statistically significant associations with ED. However, age (≥50years) (Odds ratio [OR] =1.91, 95% confidence interval [CI] = 1.28 - 2.85, P=0.001) and overweight/obesity (OR=1.81, 95%CI =1.18 - 2.77, P=0.007) were the predictors of ED.

## Discussion

This hospital-based study examined a cross-section of adult primary care patients regarding the prevalence, severity, and correlates of ED in Kano, Northwest Nigeria. It had the following main findings: An overall ED prevalence of 52.3%, with 36.0%, 14.3%, 1.5%, and 0.5% of participants having mild, mild-to-moderate, moderate and severe ED, respectively. Age, marital status, number of sexual partners, monthly income, type of exercise, history of hypertension, BMI, high blood pressure reading, use of antihypertensive and peptic ulcer disease drugs were the factors associated with ED. Age (≥50years) and overweight/obesity were the predictors of ED.

The ED prevalence of 52.3% observed in this study is similar to the 52% reported in the landmark Massachusetts Male Aging Study;^22^ it is also similar to the 55.1%, 57.4% and 58.9% found in primary care settings in Ibadan (south-west Nigeria) and the national prevalence (a multi-country survey), and a community-based study in Ogbomoso (south-west Nigeria), respectively.^8^,^10^,^11^ However, our finding is higher than the 19.8%, 34%, 41.5%, 49.4% found in similar settings in Benin (south-south Nigeria),^23^ Australia,^7^ Calabar (South-south, Nigeria),^3^ and Canada,^6^ respectively. Interestingly, our finding is lower than the prevalences of 63.6%, 80.8%, and 83% obtained in primary care settings of Egypt and Pakistan and a Nigerian psychiatric outpatient clinic.^8^,^24^ The differences in ED prevalence in the various studies could be due to the study tool used (e.g., International Index of Erectile Function [version 5] versus single National Institute of Health [NIH] consensus questionnaire) and the unique population characteristics. Despite the varying prevalences, current estimates suggest that ED remains a common health problem in all settings, including a primary care setting in Kano, Northwest Nigeria.

Furthermore, mild ED was the commonest form of ED in this study. Similar findings were reported in previous local and international studies^2^,^3^,^7^, ^9^-^12^ However, our mild ED prevalence of 36% was higher than those reported in Calabar, South-south Nigeria (16%),^3^ Osogbo, South-west Nigeria (29.4%), Ibadan, South-west Nigeria (32.6%,),^11^ Ilorin (34.3%, North-central Nigeria),^9^ and Malaysia (33.1%)^25^. However, our finding was lower than the 42.7% reported in Ogbomoso, South-west Nigeria.^10^ The varying sociodemographic characteristics, prevalence of chronic diseases like hypertension in the study populations and the ED categories analysed could explain the difference. Also, 14.3% of participants had mild-to-moderate ED, which is similar to the 14% obtained in Hong Kong.^26^ However, our finding was higher than the 8% and 8.3% reported in Calabar and Osogbo, respectively. Furthermore, only 1.5% of participants had moderate ED, which is markedly lower than 6%, and 16% reported in Calabar and Hong Kong.^3^,^26^ This difference could be due to the higher proportion of older persons (known to have higher ED prevalence) and the population with chronic medical conditions in these studies. This reason could also explain the lower severe ED prevalence of 0.5% in our study and other studies.^12^

Furthermore, previously reported factors such as age,^3^,^4^,^7^–^13^,^16^,^25^–^29^ marital status, 3,12,24,26 number of sexual partners,^9^,^30^ monthly income,^9^,^11^,^23^,^30^ type of exercise,^28^ history of hypertension,^11^–^13^,^17^,^31^ use of antihypertensive drugs,^10^,^17^,^22^,^31^ use of peptic ulcer disease drugs,^32^ BMI,^11^,^28^,^30^ and blood pressure reading^17^,^31^ were associated with ED in this study. Interestingly, the proportion of respondents with ED was higher among those with two or more sexual partners than those with one. We suspect that increased frequency of sexual activity makes them more likely to notice deficiencies in erectile function; moreover, one of the partners may observe this and point it out. Similarly, this study's higher prevalence of ED among those with higher income was also interesting. While the complete link between ED and higher income is unclear, it could be that those with higher income were more likely to be overweight or obese, have more sexual partners (as is the culture in the study area) and are older. Expectedly, age was a predictor of ED; participants aged ≥50 years had a 91% increased likelihood of having ED compared to those < 50 years of age. This finding is similar to reports from many other local and international studies. 3,4,7-13, 16,25–29 This association is attributed mainly to the higher occurrence of vascular diseases from arteriosclerosis, endothelial dysfunction, and other comorbidities with advancing age.^22^,^28^,^29^ In addition, this finding suggests the need to routinely screen for ED in this age group.

This study also found that overweight plus obesity was independently associated with ED. The participants who were overweight and obese had an 81% likelihood of having ED compared to those who were not. This has been reported by previous studies in Ibadan (Nigeria), Australia, and United States.^7^,^11^,^28^ The association between higher BMI (overweight and obesity) and ED is attributed to the presence of other strong risk factors of ED such as cardiovascular disorders, diabetes mellitus and dyslipidemia in men with higher BMI; moreover, improvements in ED had been reported following weight reduction and other lifestyle modifications.^33^,^34^ Our finding, therefore, suggests that screening for overweight and obesity, especially among the elderly, is needed for early intervention in this setting.

## Limitation of study

This was a single clinic-based study, and men presenting for medical care may differ from the general population; thus, findings may not be generalizable to the general population. Although the IIEF-5 questionnaire was used, the effect of ED stigma on participants' responses could not be eliminated. The participants' monthly income was reported, and its accuracy could not be confirmed. Screening of other correlates of ED such as glycated haemoglobin and serum testosterone was limited by financial and time constraints.

## Conclusion

The prevalence of ED was high among adult male patients, although mainly of the mild ED. Ages of ≥50 years and overweight/obesity predicted ED. This suggests the need to screen (targeting important risk factors like overweight and obesity), promptly diagnose and commence treatment of ED while referring deserving patients to specialists, and institute lifestyle modifications in this and similar settings. Similar multi-centre and population-based studies in the Northwest geopolitical region are needed in future to understand the complete picture of the problem.

## Figures and Tables

**Table 6:** Predictors of Erectile dysfunction among the participants

Variable	OR	95% CI	P-value
Age (≥50 years)	1.91	1.28 - 2.85	0.001*
Marital status (Married)	0.86	0.34 - 2.22	0.761
Number of sexual partners (≥2)	2.22	0.96 - 5.13	0.061
Monthly income (<₦18000)	0.28	0.63 - 5.01	0.276
Type of exercise (brisk walk & jogging)	0.71	0.46 - 1.08	0.109
History of hypertension (yes)	1.00	0.37 - 2.76	0.990
Antihypertensive drugs use (yes)	1.01	0.99 - 1.03	0.620
PUD medication use (yes)	1.02	0.81 - 1.04	0.581
Body mass index (overweight & obesity)	1.81	1.18 - 2.77	0.007*
Blood pressure (stage 1&2)	1.39	0.99-1.94	0.059

OR: Odds ratio; CI: Confidence interval

* Significant
